# Adhesive Performance of Pit and Fissure Sealants on Deproteinized Enamel with Different Proteolytic Agents: In Vitro Study

**DOI:** 10.3390/dj12070206

**Published:** 2024-07-04

**Authors:** Luis Francisco García-Mota, Miguel-Ángel Fernández-Barrera, Rene Garcia-Contreras, Guillermo Grazioli, Juan Eliezer Zamarripa-Calderón, José Alejandro Rivera-Gonzaga, Carlos Enrique Cuevas-Suárez

**Affiliations:** 1 Dental Materials Laboratory, Autonomous University of Hidalgo State, Circuito Ex Hacienda La Concepción S/N, San Agustín Tlaxiaca 42160, Mexico; ga204495@uaeh.edu.mx (L.F.G.-M.); miguel_fernandez10334@uaeh.edu.mx (M.-Á.F.-B.); eliezerz@uaeh.edu.mx (J.E.Z.-C.); jose_rivera10098@uaeh.edu.mx (J.A.R.-G.); 2 Nanostructures and Biomaterials Area, Interdisciplinary Research Laboratory (LII), National School of Higher Studies (ENES), Leon Unit, National Autonomous University of Mexico (UNAM), León 37689, Mexico; rgarciac@enes.unam.mx; 3 Department of Dental Materials, School of Dentistry, Universidad de la República, Montevideo 11600, Uruguay; ggrazioli@odon.edu.uy

**Keywords:** shear bond strength, deproteinizing agents, acid etch pattern, papain, bromelain, sodium hypochlorite, Tergazyme, ZYME

## Abstract

The objective of this work was to assess the efficacy of different proteolytic agents on the bond strength of pit and fissure sealants to bovine enamel. Eighty-four bovine enamel specimens were randomly assigned in groups according to the pit and fissure sealant applied (HelioSeal F or Dyad Flow). Then, the specimens were subdivided according to the proteolytic agent used (n = 7): Group 1, distilled water (control); Group 2, 10 wt.% Tergazyme^®^; Group 3, 10 wt.% ZYME^®^; Group 4, 10% papain gel; Group 5, 10% bromelain gel; and Group 6, 5.25 wt.% sodium hypochlorite. The cell viability of the proteolytic solutions was assessed through the MTT assay. The proteolytic agents were applied on the enamel surface prior to the acid-etching procedure; then, the pit and fissure sealants were placed. The micro-shear bond strength was evaluated after 24 h or 6 months of water storing at 37 °C. Representative SEM images were taken for each experimental group. The bond strength data were statistically analyzed by a three-way ANOVA test using a significance level of α = 0.05. Bromelain and papain proteolytic solutions did not exert any cytotoxic effect on the human dental pulp cells. After 24 h and 6 months of aging, for both pit and fissure sealants, sodium hypochlorite, papain, bromelain, and Tergazyme^®^ achieved statistically significant higher bond strength values (*p* < 0.05). Irrespective of the deproteinizing agent used, Dyad Flow resulted in a better bond strength after 6 months of aging. The type 1 etching pattern was identified for sodium hypochlorite, papain, and bromelain. Tergazyme^®^, papain, and bromelain demonstrated efficacy in deproteinizing enamel surfaces prior to acid etching, leading to the improved bond strength of pit and fissure sealants. Clinically, this suggests that these proteolytic agents can be considered viable alternatives to traditional methods for enhancing sealant retention and longevity. Utilizing these agents in dental practice could potentially reduce sealant failures.

## 1. Introduction

Dental caries is a disease that affects a high percentage of the global population, being a real problem of public health and one of the main causes of tooth loss [1]. Considering that this disease is associated with the development of irreversible lesions on tooth surfaces, prevention is vital, especially in early ages [2].

The placement of pit and fissure sealants is an effective method for the prevention of dental caries. Pit and fissure sealants are commonly recommended for children and adolescents as a preventive measure against dental caries. According to position statements from various scientific committees, such as the American Academy of Pediatric Dentistry [3] and the European Academy of Paediatric Dentistry [4], sealants are particularly advised for permanent molars soon after their eruption.

The effectiveness of pit and fissure sealants depends mostly on the time that the material remains on the teeth [5]. Enamel surface preparation before sealant application includes thorough cleaning and etching. Mechanical cleaning, often using a rotary brush or pumice, is routinely performed to remove plaque and debris [6]. Chemical cleaning agents, such as sodium hypochlorite, may also be used [7]; however, clinicians generally avoid high concentrations of sodium hypochlorite in pediatric patients due to its potential adverse effects [8]. To achieve an adequate retention of the pit and fissure sealant to the tooth surface, the enamel must be previously conditioned with phosphoric acid, making an etch pattern in this way [9]. The characteristics of these patterns are decisive in providing an adequate bonding surface, on which the pit and fissure sealant will be able to adhere properly; nevertheless, it is not possible to always obtain a perfect etch pattern; this is because on the dental surface, there are proteins of the acquired film that can negatively influence the bonding procedure [10].

The removal of these proteins prior to acid etching has been proven to be an effective method to enhance the shear bond strength between resinous materials and enamel [11]. For this purpose, one of the most used deproteinizing agents is sodium hypochlorite (NaClO) at 5.25% [10]. The negative effects of this agent, like the fact that it is an irritating agent to tissues, make clear the necessity to identify other chemical agents with deproteinizing capacities, which have similar effects, but with no side effects that contraindicate its use [7].

Papain is a proteolytic enzyme extracted from *Carica papaya*’s latex that has antimicrobial and anti-inflammatory properties [12]. It has been proven that the use of papain in gel presentation at a concentration of 8% to 10% is effective as a deproteinizing agent prior to acid etching, enhancing shear bond strength [13]. Another proteolytic agent, bromelain, extracted from pineapple stems, has been studied as an active substance of teeth whiteners; in this study, its proteolytic capacity was proven [14]. In addition to these, there are different enzymatic soaps, such as brands like ZYME^®^ and Tergazyme^®^, which are a combination of proteases that are able to eliminate protein deposits or biofilm from surfaces [15]. 

To the best of our knowledge, enzymatic cleaners have never been used before as deproteinizing agents for adhesive restorations; therefore, the objective of this study was to assess the efficacy of different proteolytic agents on the bond strength of pit and fissure sealants to bovine enamel. The null hypothesis was that the bond strength of two pit and fissure sealants will not be affected by the use of different proteolytic agents. 

## 2. Materials and Methods

### 2.1. Experimental Design

In this study, the bond strength of resin-based materials to bovine enamel was evaluated according to the following factors: (1) the type of pit and fissure sealant at two levels: HelioSeal F (Ivoclar-Vivadent, Schaan, Liechtenstein) or Dyad Flow (Kerr, Brea, CA, USA); (2) proteolytic agent at five levels: sodium hypochlorite, papain, bromelain, XZYME^®^ (Clean solutions, Jonesboro, AR, USA), and Tergazyme^®^ (Alconox Inc., White Plains, NY, USA); and (3) aging at two levels: 24 h and 6 months. A group without the application of a proteolytic agent was used as a control. The primary response variable was bond strength (n = 7). The sample size was estimated based on a previous study [16] that evaluated the effect of the application of a deproteinizing agent on the bond strength of a composite resin to enamel in a comparative study design with 6 independent groups, a 3.85 minimum detectable difference in means, a 1.83 standard deviation, a power of 0.8, and an a = 0.05. The sample size was calculated by using a graphing software program (SigmaPlot 14.0; Systat Software, Inc., Chicago, IL, USA). 

### 2.2. The Preparation of the Proteolytic Agent Solutions

Experimental solutions containing papain, bromelain, ZYME^®^, and Tergazyme^®^ were prepared. Papain and bromelain were obtained from Sigma-Aldrich, and they were used as received. Briefly, known amounts of the different proteolytic agents were weighted using an analytic balance and dissolved in distilled water to produce 10 wt.% solutions. The solutions were agitated using an agitation plate until the complete dissolution of the proteolytic agent. Then, the solutions were stored in glass vials and refrigerated. The materials were applied immediately after their preparation. For sodium hypochlorite, a 5.25 wt.% commercial solution was used (Viarzoni-T, Viarden™ Lab, Mission, TX, USA). 

### 2.3. Cell Viability of Proteolytic Agent Solutions

The cytotoxicity test was performed according to ISO specification 10993-5:2009 [17]. Human dental pulp stem cells were in modified eagle’s minimal essential medium (MEM, Sigma-Aldrich, St Luis, MO, USA) supplemented with 10% fetal bovine serum (FBS, Sigma-Aldrich), 10,000 IU/mL penicillin, 100 mg/mL streptomycin (Penstrep, Sigma-Aldrich), and 1% Glutamax (Life Technologies, Gibco, Grand Island, NE, USA). Cells were incubated at 37 °C in a humidified atmosphere of 5% CO_2_. 

The primary cell culture of human dental pulp stem cells (hDPSCs) was obtained from the cell bank at the Interdisciplinary Research Laboratory, Nanostructures and Biomaterials Area of the ENES León, UNAM. The cell isolation protocol, registered under number CE_16 004_SN, was approved by the bioethics committee. This approval ensures adherence to national health laws and the Helsinki Declaration, including informed consent procedures obtained prior to the extraction procedure. The cells were established and characterized as previously described [18]. Initially, the cryopreserved cells were thawed by placing each vial in a cell incubator for 5 to 10 min. After thawing, the cells were transferred to 10 cm culture plates (Corning Costar^®^, Nagog Park Acton, MA, USA) containing MEM cell culture medium supplemented with 10% fetal bovine serum (FBS), 1% glutamine (Sigma-Aldrich), and 1% antibiotics (PenStrep, Sigma-Aldrich). The cells were incubated at 37 °C with 5% CO_2_. The culture medium was refreshed every two days until the cells achieved over 80% confluence.

Cells (2 × 10^5^ cells/mL) were inoculated into a 96-microwell plate and incubated for 48 h to achieve complete adherence to the culture dish. The cells were contacted with the proteolytic agents and incubated for another 24 h. The number of viable cells was determined by the MTT method (Sigma-Aldrich). The culture medium was replaced with MTT (0.2 mg/mL) dissolved in MEM medium, and the cells were incubated for 7 h at 37 °C, 95% humidity and 5% CO_2_. The formazan products were then dissolved with dimethyl sulfoxide (100 μL, DMSO, Karal, León de los Aldama, Mexico), and absorbance at 570 nm was determined using a microplate reader (Thermo Scientific, Waltham, MA, USA).

### 2.4. Specimen Preparation

Eighty-four bovine incisors were obtained from the city’s abattoir and stored in 0.1% thymol solution for 1 week. After cleaning with distilled water, the root was sectioned, and the crowns were embedded in impression compound. Square-shaped enamel specimens (10 mm × 8 mm) were cut from the buccal surface of the teeth using a low-speed diamond blade. The specimens were placed in PVC molds and embedded in self-curing acrylic resin, and then the enamel was treated with a 600-grit silicon carbide sandpaper to obtain flat and standardized surfaces. Then, all specimens were subjected to a process intended to induce the formation of the acquired film. The fresh natural saliva used was collected from a healthy volunteer, who was instructed to brush her teeth and not to eat for 1 h before the experiment. For the acquired film formation, a drop of saliva was applied on the enamel surface with a microbrush and then stored in a desiccator at 37 °C for 24 h [19].

### 2.5. Deproteinization Process

After the induction of the formation of the acquired film, the specimens were randomly allocated to the following groups (n = 7): distilled water (control), 10 wt.% XZYME^®^, 10 wt.% Tergazyme^®^, 10 wt.% papain, 10 wt.% bromelain, and 5.25 wt.% sodium hypochlorite. The proteolytic agents were applied on the tooth surface with the help of a microbrush applicator and actively applied for 15 s, and then the surface was rinsed with water and cleaned with air. 

### 2.6. Restoration Procedures and Shear Bond Strength

All specimens were randomly assigned into 2 main groups according to the pit and fissure sealant used: Helioseal F or Dyad Flow. For the bonding procedures, the enamel sites were etched with 37% phosphoric acid (Etching Gel) (Eco-etch, Ivoclar-Vivadent) for 30 s, rinsed thoroughly for 20 s, and dried with a mild oil-free air stream. Then, a silicon mold (0.5 mm thickness) with two cylindrical orifices (1.5 mm diameter) was placed on the surface of the etched enamel. Each orifice was filled with HelioSeal F or Dyad Flow, and after that, the material was light-cured according to the manufacturer’s indications with a LED light-curing unit (Bluephase N, Ivoclar-Vivadent) with an intensity of 1000 mW/cm^2^, resulting in two pit and fissure sealant resin cylinders for each enamel specimen. After composite cylinder fabrication, enamel specimens were stored in distilled water at 37 °C. After 24 h of aging, half of the composite samples were tested for shear bond strength, while the other half, which was stored under the same conditions, was tested after 6 months. A stainless steel wire (0.2 mm diameter) was looped around each cylinder and aligned with the bonded interface for the shear bond strength test. A universal testing machine with a load cell of 1000 N (Instron 4465, Norwood, MA, USA) was used to measure the SBS at a crosshead speed of 0.5 mm/min. The bond strength was calculated considering the measured debonding force and the size of the bonding area. Failures were observed under a magnification of up to 40× using a stereomicroscope to determine the modes: adhesive, mixed, or cohesive.

### 2.7. Scanning Electronic Microscopy Observation

Three enamel specimens were prepared for each experimental and control group. The procedures of the induction of the acquired film and deproteinization were performed as described above. All samples were placed on stubs for gold sputtering and then coated with gold and prepared for surface SEM analysis. The observation zones were randomly selected at 3 different sites for each specimen. Micrographs were obtained at 100X magnification. 

### 2.8. Statistical Analysis

The sample size for each test had a power of at least 0.8 at a significance level of 0.5. Data normality was verified using Shapiro–Wilk’s test and the homoscedasticity using Levene’s test. The statistical analyses were carried out according to the different experimental designs at a significance level of a = 0.05. The statistical tests were conducted using Sigma Plot 12.0 software. The cell viability was analyzed by a one-way ANOVA. The bond strength data were statistically analyzed by a three-way ANOVA test—the factors were as follows: type of pit and fissure sealant (HelioSeal F or Dyad Flow); proteolytic agent (sodium hypochlorite, papain, bromelain, XZYME^®^, and Tergazyme^®^); and aging (24 h and 6 months). Multiple comparisons were performed through Tukey’s post hoc test. 

## 3. Results

**Figure 1:**
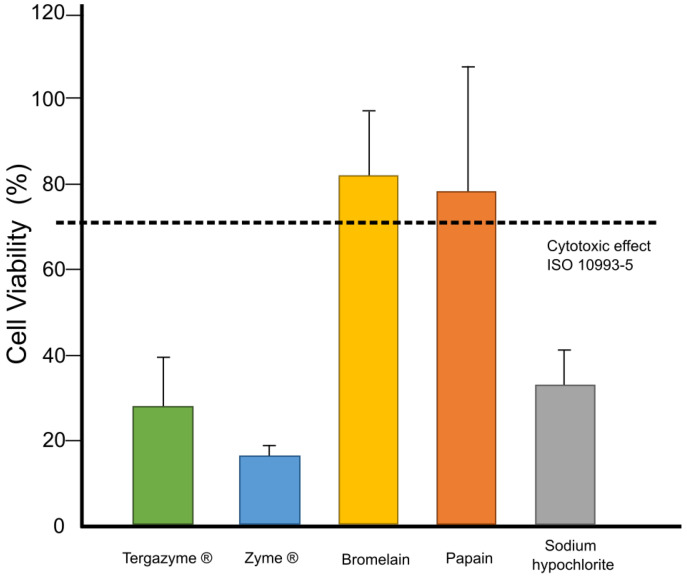
The cell viability of human dental pulp stem cells after the exposure of proteolytic solutions. Different lowercase letters represent statistically significant differences between groups (*p* < 0.05). The value indicated by the dotted line at 70% corresponds to the minimum value of cell viability established by the standard to consider a material as non-cytotoxic [20].

Figure 1 shows the results from the cell viability test of the proteolytic solutions prepared. According to the analysis, there were statistically significant differences in the mean of the cell viability of the materials tested (*p* < 0.001). Bromelain and papain proteolytic solutions did not exert any cytotoxic effect on the human dental pulp cells, both materials achieved >70% cell viability, and there were no statistically significant differences between these materials (*p* = 0.948). On the other hand, Tergazyme^®^, Zyme^®^, and sodium hypochlorite were cytotoxic to dental pulp cells.

Table 1 shows the three-way ANOVA. According to this, the factors pit and fissure sealant and proteolytic agent were statistically significant (*p* < 0.001), while the factor aging was not statistically significant (*p* = 0.854). On the other hand, the interactions pit and fissure sealant * proteolytic agent and pit and fissure sealant * aging were statistically significant (*p* ≤ 0.047).

Table 2 shows the average and standard deviation for the shear bond strength (MPa) after 24 h of aging. 

For the factor pit and fissure material, only statistically significant differences were found between the materials when the papain proteolytic agent was used. Regarding the factor proteolytic agent, for both pit and fissure sealants, sodium hypochlorite, papain, bromelain, and Tergazyme^®^ achieved statistically significant higher values than the control group (*p* < 0.05). Only Zyme^®^ resulted in similar values to the control group (*p* > 0.05). 

Table 3 shows the average and standard deviation (MPa) of the shear bond strength after 6 months of aging. When considering the factor pit and fissure sealant, statistically significant higher values were observed for Dyad Flow when papain, bromelain, and Zyme^®^ were used (*p* > 0.05). In addition, when analyzing the factor proteolytic agent, for both pit and fissure materials, statistically significant higher values were observed when sodium hypochlorite, papain, bromelain, and Tergazyme^®^ were used.

Table 4 shows the comparison of the bond strength values between 24 h and 6 months of aging for each pit and fissure sealant considering the proteolytic agent used. For Helioseal F, bond strength values remained stable for sodium hypochlorite, papain, and Tergazyme^®^. On the other hand, for Dyad Flow, there were no statistically significant differences between 24 h and 6 months when sodium hypochlorite, papain, bromelain, and Zyme^®^ were used.

**Figure 2:**
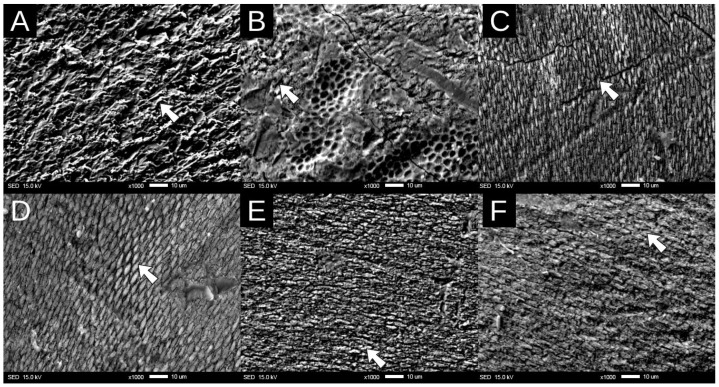
SEM images at 1000× showing the etching pattern after the application of (**A**) Tergazyme^®^, (**B**) Zyme^®^, (**C**) bromelain, (**D**) papain, and (**E**) sodium hypochlorite proteolytic agents. (**F**) is showing the control group with distilled water. The arrows show enamel prisms.

Representative SEM micrographs are shown in Figure 2. The type 1 pattern was identified for sodium hypochlorite, papain, and bromelain. On the other hand, the type 2 pattern was identified for Tergazyme^®^. Finally, a type 3 pattern was identified for Zyme^®^. 

## 4. Discussion

In this work, the bond strength of two different pit and fissure sealants on deproteinized bovine enamel with different proteolytic agents was assessed. According to the results, sodium hypochlorite, papain, bromelain, and Tergazyme^®^ achieved statistically significant higher bond strength values than the control group; only Zyme^®^ was not able to increase the bond strength values. Considering this, the null hypothesis tested in this study was partially rejected. 

In this study, both HelioSeal F and Dyad Flow were used as pit and fissure sealants. HelioSeal F, a light-curing, fluoride-releasing sealant, relies on prior acid etching to enhance mechanical bonding. Dyad Flow, although possessing self-etching properties, also underwent an additional acid-etching step in our experimental protocol. This uniform etching approach ensured consistent surface preparation, allowing us to focus on the effects of the proteolytic agents. The enhanced bond strengths observed in both sealants underscore the importance of thorough enamel etching, which facilitates the optimal sealant retention and efficacy.

Pit and fissure sealants’ success depends on the retention it has with dental structure; this way, dentobacterial plaque accumulation will be prevented [21]; to obtain this retention, modifying the enamel surface is necessary, through acid etching, in order to dilute hydroxyapatite crystals, making an etch pattern in this way [9]. This pattern is better if the acquired film is eliminated, enhancing bond strength between pit and fissure sealants and the enamel. To eliminate the acquired film, sodium hypochlorite has been used [22]; however, the risk of this agent for the patient, especially for pediatric dentistry, pushes us to study alternatives for this use. 

Nowadays, sodium hypochlorite at 5.25% is considered as the gold standard for the enamel deproteinization technique [22]. Justus et al. indicated that shear bond strength was significantly enhanced with the application of sodium hypochlorite. The findings of our research are in agreement with the aforementioned work, since for HelioSeal F and Dyad Flow, when compared to the control group, there was a significant increase when sodium hypochlorite was used as a deproteinizing agent. This result was corroborated with the images obtained by SEM, where it is clearly visible that the use of sodium hypochlorite promoted a type 1 etching pattern. It is worth mentioning that this pattern was also present in the control group; however, in this case, such a pattern was not as clearly visible as in the sodium hypochlorite group. One of the possible reasons that promoted the formation of the type 1 etching pattern was the elimination of the acquired film by sodium hypochlorite [10].

Papain was introduced in 2003 into dentistry for the chemical selective removal of caries [12]. This enzyme was also used as a deproteinizing agent before bonding procedures [13]. Actually, Pithon et al. demonstrated that the best concentration for the removal of the acquired film is at 10% [12]. This was the reason why this research used this concentration for the fabrication of the proteolytic solution. According to the results obtained, papain was effective in increasing the bond strength of both pit and fissure sealants tested. This enzyme has the ability to achieve proteolysis in immunoglobulins that are present in the acquired pellicle [23]. Papain can hydrolyze IgG into two main fragments (Fab and Fc) and one minor (Fc’). These fragments of IgG were found first in rabbit IgG and later corroborated in human IgG [24]. Papain has a proteolytic effect in IgM, resulting in different peptides that lead to two fragments that are resistant to further degradation called Fabµ y Fcµ, due to their resemblance to the remains of IgG [24]. This ability was ratified by the image obtained by SEM, which can be appreciated for the type 1 etching pattern, which is the ideal for the adhesion of resin-based materials [5].

Another deproteinizing agent of natural origin used in this study was bromelain, extracted from pineapple stems [11]. The results obtained in this study proved that this enzyme showed similar proteolytic properties to papain; actually, there were no statistically significant differences in the bond strength between bromelain, papain, and sodium hypochlorite. These results agree with those obtained by Ribeiro et al. that demonstrated that bromelain has proteolytic capacity on bovine enamel [14]. This enzyme has the ability to break the peptide bonds of IgG, resulting in two great fragments, Fab of a molecular weight of 51,000 gr/mol and Fc of 48,000 g/mol. Thus, we can deduce that these proteolytic agents work in a very similar way [25]. As occurred with papain and sodium hypochlorite, the application of bromelain for enamel deproteinization resulted in a type 1 etching pattern, which, as mentioned before, is ideal for bonding [10].

This study evaluated the use of enzymatic agents as deproteinizing agents. Tergazyme^®^ is used to remove biofilm and protein deposits [15], and this agent has not been studied before as a deproteinizing agent. The results obtained in this work showed that the bond strength values obtained with this enzymatic cleaner were similar to those obtained with sodium hypochlorite. According to the MSDS provided by the manufacturer, Tergazyme^®^ contains sodium tripolyphosphate and sodium alkylbenzene sulfonate, which are mainly used as surfactants and detergents [26]. These agents have been proven to effectively act as proteolytic agents [11] which can help to explain the results of this study. Also, according to the SEM analysis, the use of Tergazyme^®^ as a deproteinizing agent yielded type 2 etching patterns, which can favor the adhesion performance of resin-based materials [10].

ZYME^®^, another enzymatic agent used in this study, contains ethylenediaminetetraacetic acid (EDTA). EDTA is a chelating agent that has a main clinical use as an irrigant in endodontics [27]. Also, this compound has been used as dentin conditioner before bonding procedures [28]. In fact, pre-conditioning with EDTA showed similar bond strength values and enamel etching patterns for adhesive systems in fluorotic enamel [29]. Since EDTA is an acid, it is possible that, together with the application of phosphoric acid, an over-etched dental surface could be the result [30]. This could be confirmed with the type 3 etching pattern observed in the SEM micrographs. It has been previously demonstrated that an over-etched surface produces greater surface roughness but does not necessarily result in significantly higher bond strength values; in fact, in this study, the bond strength of enamel deproteinized with ZYME^®^ was similar to the control.

Finally, an analysis was performed to evaluate the bond strength stability of the materials according to the proteolytic agent used. For both pit and fissure sealants, the use of a proteolytic agent helped to maintain the bond strength values after aging. This result makes it clear that the use of proteolytic agents could lead to a stronger and more durable bond between a resin-based material and enamel, which highlights the importance of deep and proper cleaning before any bonding procedure [31]. Surprisingly, for Dyad Flow, the use of proteolytic agents increased the bond strength after aging. The increase in the bond strength of a self-adhesive material has been observed before [32], and it has been related that this behavior is due to an increase in the degree of conversion of the material after aging and also due to the formation of stable hybrid layers over time [33].

The SEM analyses demonstrated that sodium hypochlorite, papain, and bromelain predominantly produced a type 1 etching pattern, characterized by a honeycomb-like appearance. This pattern indicates a preferential dissolution of the enamel prism cores, leading to a retentive surface that may enhance the micromechanical bonding of the sealant [9]. On the other hand, Tergazyme^®^ yielded a type 2 etching pattern, characterized by the partial removal of the prism peripheries while leaving the cores relatively intact [34]. This pattern may suggest a different interaction between the proteolytic agent and the enamel, potentially affecting the bond strength differently compared to type 1. Finally, the use of Zyme^®^ resulted in a type 3 etching pattern, which involves an irregular and less distinct removal of enamel structures. This pattern could indicate a more generalized etching effect that might influence the bonding properties of the sealant [34].

The cell viability of the proteolytic agent solutions was assessed as secondary outcome. The results showed that hypochlorite sodium has a cytotoxic effect on human dental pulp cells, and this is related to its oxidizing capacity and the pH of the solution [8]. The same effect was observed for Tergazyme^®^ and ZYME^®^, where the main constituents, tripolyphosphate, sodium alkylbenzene sulfonate, and EDTA, have been proven to possess marked cytotoxic effects [35]. On the other hand, papain and bromelain were well tolerated by the cell line tested, promoting a cell viability above 70% in both cases. These results are not surprising since these enzymes have been used as wound-healing materials and they possess anti-inflammatory behavior [36]. 

In this study, bovine enamel was used instead of human enamel. Bovine enamel is commonly used as a substitute for human enamel in dental research due to its availability and similarity in composition and structure [37]. However, there are notable differences between bovine and human enamel that must be considered; first at all, bovine enamel has a more regular prism structure compared to human enamel. This can influence how bonding agents interact with the enamel surface. Also, bovine enamel tends to be harder and thicker than human enamel, which could affect the penetration of etching agents and the adhesion of sealants. Although similar, there are slight variations in the mineral content between bovine and human enamel, which may influence the bonding process [38]. Despite these differences, bovine enamel is considered a valid model for preliminary studies. It allows for standardized comparisons under controlled conditions, which are essential for the initial evaluation of new materials and techniques [39].

Regarding the bond strength used in this study, the shear bond strength (SBS) was used for several reasons; the SBS test is relatively simple to perform and highly reproducible, making it a standard method for evaluating the bonding performance of dental materials. The SBS test mimics the forces that dental restorations and sealants would encounter in the oral environment, providing clinically relevant information about their performance [40]. Despite these advantages, the SBS test may not accurately represent the complex stress distribution seen in clinical settings. It applies a uniform load, which is not always reflective of the multi-directional forces present in the oral cavity, and differences in surface preparation techniques can significantly influence SBS results, potentially affecting the comparability of studies [41]. To address these limitations, future studies could incorporate additional testing methods, such as micro-tensile bond strength tests, which provide a more comprehensive understanding of the bonding performance and failure modes. 

One of the primary limitations of this study is the use of flat enamel surfaces for evaluating the bond strength of pit and fissure sealants. While flat surfaces allow for standardized testing conditions and reproducibility, they do not fully replicate the complex morphology of occlusal pits and fissures found in clinical situations. Other limitations could be found in this in vitro study. First at all, the in vitro design limits the extrapolation of the results to a clinical scenario; further well-designed randomized clinical studies must be performed to prove the beneficial effect on the retention of pit and fissure sealants placed with the deproteinizing technique. Also, proteolytic agents could be formulated in the form of gel, to gain better control of their placement. Finally, the effect of these proteolytic agents on fluorotic or hypomineralized enamel could be tested too.

## 5. Conclusions

The use of Tergazyme^®^, papain, and bromelain as deproteinizing agents before the acid-etching procedures yielded higher and stable bond strength values of two different pit and fissure sealants to healthy enamel. Despite this, only papain and bromelain were considered non-cytotoxic, and therefore, these proteolytic agents are promising substitutes of sodium hypochlorite to be used as deproteinizing agents before the bonding procedures of pit and fissure sealants to achieve higher bond strength values. Clinically, this suggests that these proteolytic agents can be considered viable alternatives to traditional methods for enhancing sealant retention and longevity. Utilizing these agents in dental practice could potentially reduce sealant failures and enhance preventive measures against dental caries in occlusal surfaces.

## Figures and Tables

**Table 1:** Three-way ANOVA for pit and fissure sealant, proteolytic agent, and aging.

Source of Variation	DF	SS	MS	F	*p*
Pit and fissure sealant	1	1002.853	1002.853	46.572	<0.001
Proteolytic agent	5	1028.64	205.613	9.549	<0.001
Aging	1	0.736	0.736	0.0342	0.854
Pit and fissure sealant × Proteolytic agent	5	252.246	50.449	2.343	0.047
Pit and fissure sealant × aging	1	539.557	539.557	25.057	<0.001
Proteolytic agent × aging	5	33.86	6.772	0.314	0.930
Pit and fissure sealant × Proteolytic agent × aging	5	8.826	1.765	0.082	0.995
Residual	96	2067.215	21.533		
Total	119	4933.359	41.457		

DF: degree of freedom; SS: sum of squares; MS: mean square; F: statistic F; *p*: *p*-value.

**Table 2:** Shear bond strength as a function of the pit and fissure sealant and the proteolytic agent after 24 h of aging.

Group	HelioSeal F	Dyad Flow *
Control group	8.128 (3.22) ^b,A^	8.728 (8.32) ^b,A^
Tergazyme^®^	15.342 (6.39) ^a,A^	12.924 (1.88) ^a,A^
Zyme^®^	11.246 (3.73) ^b,A^	12.407 (6.08) ^ab,A^
Papain	12.318 (3.36) ^a,B^	20.500 (2.257) ^a,A^
Bromelain	14.200 (4.27) ^a,A^	14.240 (3.08) ^a,A^
Sodium hypochlorite	17.094 (4.72) ^a,A^	18.824 (2.48) ^a,A^

Capital letters indicate differences between pit and fissure materials for each proteolytic agent. Lowercase letters indicate differences between proteolytic agents within each pit and fissure sealant. * Data analyzed using Kruskal-Wallis test.

**Table 3:** Shear bond strength as a function of the pit and fissure sealant and the proteolytic agent after 6 months of aging.

Group	HelioSeal F	Dyad Flow *
Control group	3.92 (0.84) ^b,B^	14.92 (4.43) ^b,A^
Tergazyme^®^	15.09 (5.74) ^a,A^	18.69 (2.86) ^ab,A^
Zyme^®^	3.55 (2.01) ^b,B^	14.61 (5.87) ^b,A^
Papain	9.03 (3.19) ^a,B^	22.41 (3.43) ^ab,A^
Bromelain	5.01 (1.90) ^ab,B^	18.36 (1.41) ^a,A^
Sodium hypochlorite	14.93 (4.92) ^a,A^	22.68 (3.99) ^a,A^

Capital letters indicate differences between pit and fissure materials for each proteolytic agent. Lowercase letters indicate differences between proteolytic agents within each pit and fissure sealant. * Data was analyzed using Kruskal-Wallis test.

**Table 4:** Shear bond strength as a function of the aging time for each pit and fissure sealant.

**Helioseal F**	**24 h**	**6 Months**
Control group	8.128 (3.22) ^A^	3.92 (0.84) ^B^
Tergazyme^®^	15.342 (6.39) ^A^	5.09 (5.74) ^A^
Zyme^®^	11.246 (3.73) ^A^	3.55 (2.01) ^B^
Papain	12.318 (3.36) ^A^	9.03 (3.19) ^A^
Bromelain	14.200 (4.27) ^A^	5.01 (1.90) ^B^
Sodium hypochlorite	17.094 (4.72) ^A^	14.93 (4.92) ^A^
**Dyad Flow**	**24 h**	**6 months**
Control group	8.728 (8.32) ^B^	14.92 (4.43) ^A^
Tergazyme^®^	12.924 (1.88) ^B^	18.69 (2.86) ^A^
Zyme^®^	12.407 (6.08) ^A^	14.61 (5.87) ^A^
Papain	20.500 (2.257) ^A^	22.41 (3.43) ^A^
Bromelain	14.240 (3.08) ^A^	18.36 (1.41) ^A^
Sodium hypochlorite	18.824 (2.48) ^A^	22.68 (3.99) ^A^

Capital letters indicate differences between 24 h and 6 months of aging for each proteolytic agent.

## Data Availability

The data that support the findings of this study are available from the corresponding author upon reasonable request.
